# Real‐time imaging of sodium glucose transporter (SGLT1) trafficking and activity in single cells

**DOI:** 10.14814/phy2.13062

**Published:** 2017-02-13

**Authors:** Chiara Ghezzi, Guillaume Calmettes, Pauline Morand, Bernard Ribalet, Scott John

**Affiliations:** ^1^ Departments of Physiology and Medicine and the Cardiovascular Research LaboratoryDavid Geffen School of Medicine at UCLALos AngelesCalifornia

**Keywords:** Endocytosis, lysosome, proteasome, SGLT1

## Abstract

The processes controlling targeting of glucose transporters to apical and basolateral membranes of polarized cells are complex and not‐well understood. We have engineered SGLT1 and GLUT4 constructs linked to fluorescent proteins to highlight the differences in transporter expression and trafficking, in real time, in different cell types. Activity was assessed in parallel using a FRET glucose sensor. In COS cells and HEK cells, SGLT1 was distributed between the plasma membrane and intracellular compartments, but there was little expression in CHO cells. Trafficking was investigated using the lysosome inhibitors NH_4_Cl (10 mmol/L) and chloroquine (150 *μ*mol/L) and the proteasome inhibitors MG‐262 (1 *μ*mol/L) and lactacystin (5 *μ*mol/L). Lysosome inhibitors caused SGLT1 accumulation into intracellular bodies, whereas proteasome inhibitors induced SGLT1 accumulation in the plasma membrane, even in CHO cells. Our data suggest that a fraction of SGLT1 is rapidly degraded by lysosomes and never reached the plasma membrane; another fraction reaches the membrane and is subsequently degraded by lysosomes following internalization. The latter process is regulated by the ubiquitin/proteasome pathway, acting at a late stage of the lysosomal pathway. Using the cholesterol inhibitor M*β* CD (3 mmol/L), a dominant negative dynamin (K44A) and caveolin, we showed that SGLT1 internalization is lipid raft‐mediated, but caveolin‐independent. In contrast, GLUT4 internalization is dynamin‐dependent, but cholesterol‐independent. The physiological relevance of these data is discussed in terms of differential membrane compartmentalization of the transporters and expression under stress conditions.

## Introduction

In intestinal absorptive cells and epithelial cells of the kidney, glucose (and galactose) is absorbed from the lumen, against its gradient, by the electrogenic Na/glucose co‐transporter SGLT located in the brush‐border or apical membrane – among the many isoforms, SGLT1 is most abundant in the small intestine, whereas SGLT2 is almost exclusively found in kidney (Wright et al. 2011). The energy for uphill sugar transport is provided by the sodium electrochemical potential gradient. The sugar that accumulates in the cell, due to SGLT‐dependent transport, exits down its gradient, across the basolateral membrane and into the blood, via the facilitative glucose transporter GLUT (Wright et al. 2011). Thus, the location of SGLT at the apical membrane and of GLUT at the basolateral membrane is critical for the unidirectional transport of glucose across epithelia. It remains that little is known about the processes that target the two transporters to their respective membrane compartments.

The density of proteins in the plasma membrane involves a balance between insertion and retrieval. One or both of these processes may be regulated to increase or decrease the level of protein in the plasma membrane. In most cells, insertion involves exocytotic vesicles that bud from the trans Golgi network (TGN), or vesicles that recycle from the early endosome. In polarized epithelial cells, protein insertion is more complex since some proteins are targeted to the basolateral membrane, while others end up in the apical membrane. The targeting to the basolateral membrane is relatively straightforward and involves exocytotic vesicles originating from the TGN. In contrast, targeting to the apical membrane may involve a direct route from the TGN to the apical membrane or a transcytosis pathway where proteins transit by the basolateral membrane before reaching the apical membrane (Rodriguez‐Boulan et al. 2005; Lakkaraju and Rodriguez‐Boulan 2007; Sato et al. 2007).

Retrieval of proteins from the plasma membrane remains the subject of many studies. Three broad categories have been identified for internalization of membrane‐bound proteins, extracellular particles, or membrane components in general. These categories involve lipid raft, clathrin‐coated pits, or phagocytosis. Lipid raft‐mediated endocytosis may be further divided into caveolin‐dependent and independent pathways (Le Roy and Wrana 2005; Ivanov 2008; El‐Sayed and Harashima 2013; Parton and del Pozo 2013). Methyl‐*β*‐cyclodextrin (M*β*CD), which depletes cholesterol from membrane's rich domains, is widely used to study membrane proteins that are internalized via caveolin‐dependent or ‐independent pathways. In this case, inhibition of lipid raft‐dependent endocytosis by M*β*CD increases insertion of the selected proteins in the plasma membrane (Roepstorff et al. 2002; Ivanov 2008). Accordingly, based on the observation that M*β*CD decreases both the activity and insertion of SGLT1 into the apical membrane, it has been suggested that cellular cholesterol stabilizes SGLT1 into the apical membrane (Suzuki et al. 2006). The GTPase dynamin regulates both the clathrin and caveolin‐dependent pathways, but more importantly, does not regulate the lipid raft endocytotic pathway, which is caveolin‐independent (Achiriloaie et al. 1999; Yao et al. 2005; El‐Sayed and Harashima 2013). A dominant negative dynamin (K44A) has been identified that prevents internalization of membrane‐bound proteins that use caveolin‐ and clathrin‐dependent pathways for retrieval (Oh et al. 1998; El‐Sayed and Harashima 2013). We have used both M*β*CD and dynamin K44A to characterize SGLT1 and GLUT4 internalization and found that SGLT1 internalization is lipid raft mediated, but caveolin‐independent, while GLUT4 endocytosis is dynamin‐dependent and, more importantly, M*β*CD‐independent.

Membrane‐bound proteins are degraded by the endosome/lysosome pathway, while cytosolic proteins, or misfolded proteins from the ER are degraded by the ubiquitin/proteasome pathway. However, ubiquitin also tags membrane‐bound receptors and channels such as EFGR, GHR, and connexin43 (Cx43) to facilitate their internalization and degradation by lysosomes. In this instance, proteasome inhibitors may block a late stage of the lysosomal pathway and, as a result, increase protein recycling between the early endosome and the plasma membrane (Laing et al. 1997; van Kerkhof et al. 2001; Longva et al. 2002; Alwan et al. 2003). Our experiments carried out with three different cell lines, and several lysosome and proteasome inhibitors indicate that SGLT1 expression and targeting is similar to that of Cx43. Also, as with EGFR and GHR (van Kerkhof et al. 2001; Longva et al. 2002; Alwan et al. 2003), lysosome inhibitors target SGLT1 to intracellular bodies and proteasome inhibitors increase membrane insertion of SGLT1, by inhibiting very likely the lysosomal pathway.

## Experimental Procedures

### Solutions and experimental techniques

The bath solution for cell imaging consisted of (in mmol/L) 140 NaCl, 5 KCl, 1.1 MgCl2, 2.5 CaCl_2_, 10 HEPES, with the pH adjusted to 7.2 with KOH. Glucose was added to this solution and for the 0 glucose solution, N‐methyl‐d‐glucamine (NMDG) was added to maintain the solution's osmolarity. Solutions were perfused directly over the cells using a gravity fed eight ways perfusion device (Warner Instruments, Hamden, CT) with electrically controlled solenoids (The Lee Company, Westbrook, CT). Input and output of solution volumes to the recording chamber (35 mm glass bottomed culture dish) were equilibrated to maintain constant flow rates and pressures within the recording chamber. Incubation of the cells with the various chemicals was carried out at 37°C and the imaging was done at room temperature (25°C). The cholesterol inhibitor (M*β*CD), chloroquine and other chemicals were purchased from Sigma‐Aldrich Corp. (St. Louis, MO). The proteasome inhibitor III and lactacystin were purchased from Calbiochem.

### Plasmids constructs and transfection

We obtained the FLIPglu‐700 *μ*mol/L cDNA from Dr. W.B. Frommer, GLUT4 from Dr. D. Abel, and human SGLT1 from Dr. E. Wright. The following plasmids were obtained from Addgene:Lamp1‐YFP (Addgene plasmid 1816), GFP EEA1 (Addgene plasmid 42307), Lyn‐tailed mCherry‐SEpHluorin, (Addgene plasmid 32002) – The latter had the SEphlorin removed to make the plasma membrane marker PM‐mCherry, using the N‐terminal motif of the Src family kinase Lyn (Koivusalo et al. 2010), Dynamin wt (Addgene plasmid 34684), and Dynmamin dominant negative (Addgene plasmid 34685). Caveolin was obtained from Origene. Caveolin was fused at the C‐terminus to mCherry/CFP. Human SGLT1 with insertions of YFP or CFP was generated by inserting a HindIII site at the individual amino acids of 55, 595 and subcloning into the restriction site a HindIII flanked YFP or CFP. All constructs were subcloned into the mammalian expression vectors with the CMV promoter. The IgKappa signal sequence (Invitrogen, Carlsbad, CA) was added to the N‐terminus of the fusion protein YFP/CFPSGLT1 to induce insertion of the functional construct into the plasma membrane. Transient transfections of all cell lines with all constructs were done after 1 to 2 days in culture with Lipofectamine 2000 (Invitrogen). Expression of the constructs was sufficiently high after 36–48 h to perform radioactive uptake, protein isolation for western blots, and microscopy imaging.

### FRET imaging

All cells were imaged live without fixation. Images (16‐bit) were acquired using a Nikon Eclipse TE300 microscope fitted with a 60X (N.A. 1.4) oil immersion lens (Nikon Instruments Inc., Melville, NY) and equipped with a filter cube comprising a CFP bandpass excitation filter: 436/20b, together with a longpass dichroic mirror: 455DCLP (Chroma Technology Corp, Rockingham, VT). LED's (Lumileds; San Jose, CA) were used as light sources: one emitting at 455 ± 20 nm (royal blue) and the other emitting at 505 ± 15 nm (cyan). LED's and camera exposure were controlled by MetaFluor Imaging 6.1 software (Molecular Devices Corp., Sunnyvale, CA). Ratiometric FRET measurements were monitored from the Yellow Fluorescent Protein (YFP) and Cyan Fluorescent Protein (CFP) images acquired simultaneously using a Dual View image splitter (Optical Insights, Tucson, AZ) equipped with a 505 nm long‐pass dichroic filter to separate the CFP and YFP signals, a CFP emission filter (480/30) and a YFP emission filter (535/40) (John et al. 2008, 2011). Images were captured with a Cascade 512B digital camera (Photometrics, Tucson, AZ). Exposure times were optimized in each case but varied between 300 and 500 ms and were recorded at a constant rate for each cell between 0.2 and 0.33 Hz. Many experiments lasted more than 1 h leading to a slow drift in the FRET ratio baseline in some cases.

### Standard microscopy imaging

Standard microscopy images were acquired using an Olympus IX70 inverted microscope (Olympus America Inc., Center Valley, PA) fitted with an Olympus plan apo 60×, 1.4 N.A. oil immersion objective and a cooled CCD camera (Model Quantix, Photometrics, Tucson, AZ). Imaging Workbench software was used for data acquisition and analysis. YFP (XF104‐2) and CFP (XF130‐2) filter cubes were purchased from Omega Optical Inc. Confocal images were acquired using on a Zeiss Axiovert 100 LSM inverted microscope fitted with a 60× water immersion objective (Zeiss CApochromat 63/1.2 W Corr). Zeiss Pascal 5 image software (Carl Zeiss, Inc., Thornwood, NY) was used for data acquisition and analysis.

### Immunoblot analysis

For immunoblot analyses, cells were lysed in a buffer containing 50 mmol/L Tris, pH 7.5, 1% Triton X‐100, 150 mmol/L NaCl, 10% glycerol, and 1 mmol/L EDTA. Samples were size fractionated by SDS‐Page (10%) and transferred to nitrocellulose membrane (Biorad, Hercules, CA).

Immunodetection was performed using the following primary antibodies, goat anti‐Actin (I‐19) (diluted 1:500), rabbit anti‐GFP (FL) (diluted 1:200) all purchased from Santa Cruz Biotechnology, Inc., Santa Cruz, CA. As secondary antibodies, we used rabbit anti‐goat Ig/peroxidase, goat anti‐rabbit Ig/peroxidase, and goat anti‐mouse Ig/peroxidase (all diluted 1:5000), all purchased from Sigma‐Aldrich. For quantitative analysis, revelation was performed using the Luminata Forte Western HRP Substrate as describe by the manufacturer Millipore (Temecula, CA). Chemiluminescence images were acquired using an Image Reader LAS‐3000 LCD camera (Fujifilm, Stamford, CT). Band intensities were quantified using National Institutes of Health ImageJ software.

### Quantitative analysis of SGLT1 localization

Differential Localization of SGLT1‐YFP in the cytoplasm and plasma membrane was quantified using the intensity profile method developed by ImageJ. The nuclear region was excluded when drawing the profiles. We also attempted to pick regions, from the membrane and cytoplasm, that exhibited uniform fluorescence intensity i.e., “hot spots” were excluded. To quantify the degree of membrane insertion, we first estimated the areas under the peaks of fluorescence, which correspond to membrane‐bound SGLT1. These values were then normalized to the cell area laying below the profile. Ten measurements were carried out in all cases.

### Statistical analysis

For each data set, the mean and accompanying 95% confidence intervals (CIs) are reported. The conventional percentile bootstrap‐resampling approach with 10,000 replications was used for estimating 95% CI as well as examining the significant difference between groups (effect size statistics) (Efron and Tibshirani 1991; Nakagawa and Cuthill 2007; Calmettes et al. 2012). A *P*‐value <0.05 was considered statistically significant. All analyses were performed by subroutines for bootstrapping developed in the Python programming language (van Rossum and Drake 2001), using the Numpy (Oliphant 2001) and Scipy (Jones et al. 2001) packages, based on the code we previously published (Calmettes et al. 2012).

## Results

### Generating SGLT1 constructs linked to YFP or CFP

**Figure 1:**
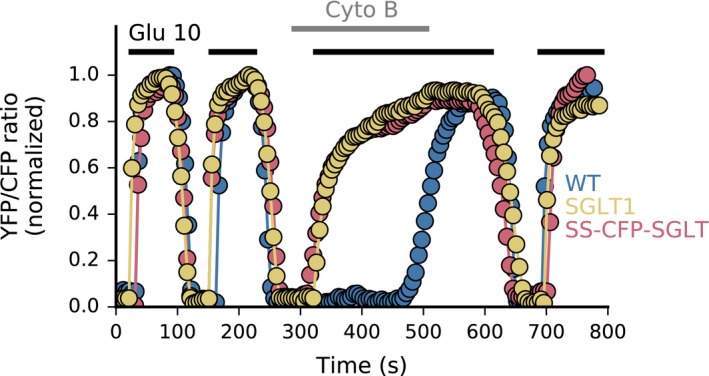
SGLT1‐dependent glucose transport in COS cells. The differences in FRET ratios in Fig 1 illustrate how overexpression of wt SGLT1 and fluorescent construct SS‐CFP‐SGLT1 modulate glucose entry in COS cells expressing the FRET‐ based glucose sensor Flip 600 *μ*mol/L. In this panel, three traces are superimposed that were obtained in three sets of experiments with COS cells expressing either wt SGLT1, SS‐CFP‐SGLT1 or no SGLT1. At the beginning of the traces, glucose entry resulting from the addition of 10 mmol/L glucose to the bath was mediated by both endogenous GLUTs and overexpressed SGLT1. In all three cases, glucose efflux following bath glucose removal was mediated via endogenous GLUTs. In the middle of the traces, 10 *μ*mol/L CytoB was added to the bath to block endogenous GLUTs. Under these conditions, addition of 10 mmol/L glucose to the bath evoked glucose entries of similar amplitude with cells expressing wt SGLT1 and SS‐CFP‐SGLT1, indicating that SS‐CFP‐SGLT1 is fully functional. The fact that there is no glucose entry in cells that do not overexpress SGLT1, suggest that there is no detectable endogenous SGLT activity in COS cells.

The regulation of GLUT insertion in the plasma membrane has been extensively studied and is reasonably well‐understood (Hou and Pessin 2007). In comparison, less is known about the regulation of SGLT insertion in the plasma membrane. We have generated several SGLT1 constructs linking the fluorophores YFP or CFP at different locations to study this process. In generating constructs, we were looking for functional and nonfunctional transporters that translocated to the membrane. The rationale behind this approach was to assess whether activity modulates transporters’ membrane insertion or retrieval. To measure function – that is glucose transport – we employ a well‐established technique, based on cell's transfection with a FRET glucose biosensor, which allows recording of glucose fluxes and metabolism in single cells (John et al. 2011; Calmettes et al. 2013). Out of the many constructs that we generated, we focused on one active and one inactive construct, which had both very good level of expression and were translocating to the plasma membrane. Many of the constructs that we generated were nonfunctional and the one we picked for our study had the fluorophore inserted at residue 595 (SGLT1‐595YFP). Generating a functional transporter with CFP or YFP was challenging, and we found that linking the fluorophore to the N terminus, which can be altered and still allow for normal protein folding (Faham et al. 2008), yielded functional transporter activity. To further facilitate protein insertion into the plasma membrane, we inserted a Signal Sequence in front of the fluorophore at the N terminus (see Materials and Methods). This construct denoted SS‐CFP‐SGLT1, had a five residues linker between CFP and SGLT1, and transported glucose as the wt SGLT1 (Fig. 1). To validate our functional assay, we also generated a similar construct with SGLT2 (SS‐CFP‐SGLT2) that we overexpressed in COS cells. Data in Fig. S1 show that the SGLT2 construct generated very little glucose uptake compared to SGLT1 under the same conditions. The very low transport activity of SGLT2 compared to SGLT1 is consistent with previous reports (Hummel et al. 2011).

### Translocation of SGLT1 and GLUT4 to the plasma membrane of HEK, COS, and CHO cells

**Figure 2:**
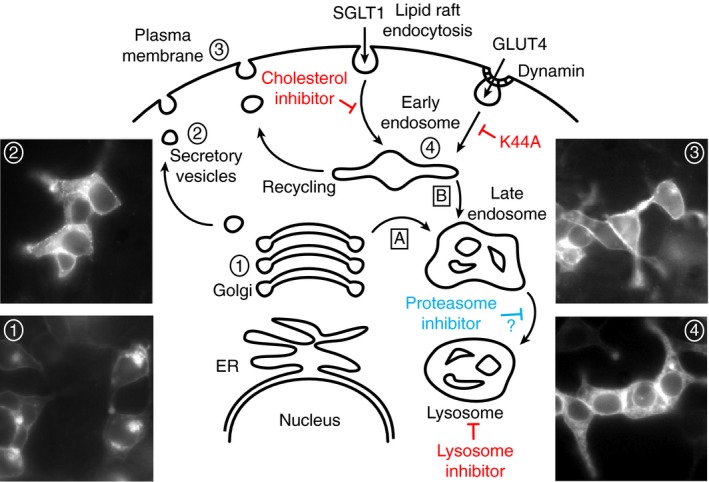
SGLT1 trafficking uses a classical pathway in HEK and COS cells. Depending on the experimental conditions, our data show that SGLT1‐YFP may be found in the trans Golgi network (TGN) (1), vesicles (2), the plasma membrane (3), or endosome (4). These images suggest that insertion and retrieval of SGLT1 in and out of the membrane follows a classical pathway. This diagram also specifies the modulator of endocytosis and degradation that were used and their potential sites of action. The letter “A” identifies the trafficking of proteins from the TGN to lysosome and “B” identifies the trafficking of endocytosed proteins from the plasma membrane to lysosome. The mode of action of the GTPase dynamin is also identified in this diagram.

Trafficking of SGLTs to the plasma membrane in polarized cells is poorly understood. To study this process, we transfected SGLT1 linked to YFP or CFP into HEK, COS, and CHO cells. Experiments were carried out with both SGLT1‐595YFP and SS‐CFP‐SGLT1. Trafficking of these two constructs was qualitatively the same and we will use the denotation SGLT1‐YFP/CFP in the rest of the text to designate the two constructs. In considering SGLT1 trafficking, we assumed that trafficking of SGLT1 in these cell lines follows a classical pathway, wherein after synthesis, the protein moves to the trans Golgi network (TGN) and from there to the plasma membrane via exocytotic vesicles (Fig. 2). Insertion is then balanced by retrieval mechanisms, which target the protein to the lysosomal pathway or back to the plasma membrane via a recycling process, involving the early endosome. Figure 2 shows that SGLT1‐YFP is observed at all of these steps – that is, TGN, exocytotic vesicles, plasma membrane, and endosome, supporting the hypothesis that trafficking of SGLT1 uses a conventional pathway. In this study, we focused our attention on the endocytosis and degradation processes. There are two main of endocytotic pathways, which are either mediated via clathrin coated pit or lipid raft; the lipid raft‐mediated pathway may be further divided into caveolin‐dependent or ‐independent pathways. Both the clathrin‐ and caveolin‐dependent pathways are modulated by the GTPase dynamin that constricts and pinches the endocytotic vesicles from the membrane (Henley et al. 1998; El‐Sayed and Harashima 2013; Parton and del Pozo 2013). To characterize SGLT1‐YFP/CFP internalization, we used two approaches. First, we used the dynamin‐dominant negative K44A, which prevents pinching off of endocytotic vesicles and causes accumulation of the membrane vesicle's content in the plasma membrane (Oh et al. 1998; El‐Sayed and Harashima 2013). Second, we used the cholesterol inhibitor M*β*CD, which prevents endocytosis of cholesterol‐dependent vesicles.

**Figure 3:**
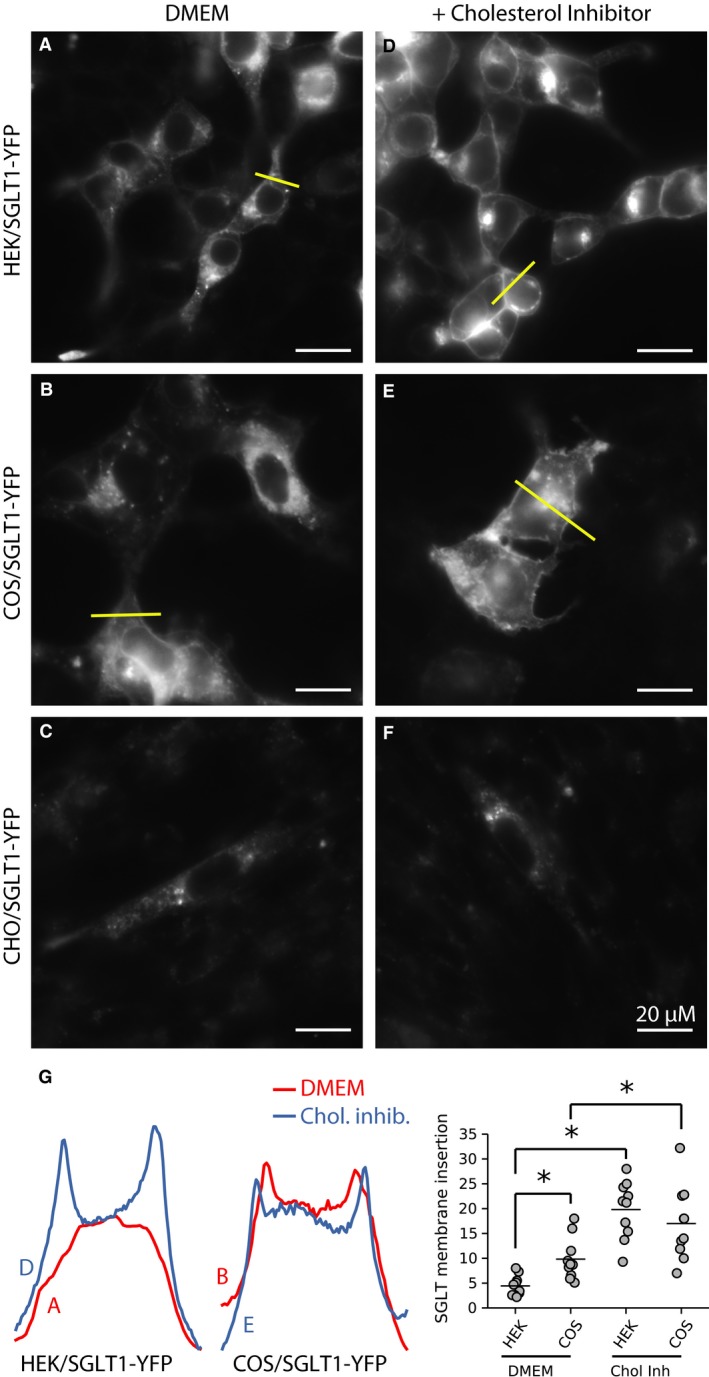
The cholesterol inhibitor M*β* CD enhances insertion of SGLT1 in the plasma membrane. In HEK cells (A) and COS cells (B), and under normal culture conditions (DMEM), SGLT1‐YFP is primarily located inside the cells in small vesicles and in larger structures identified as endosomes. In contrast, there is almost no SGLT1 expression in CHO cells. An image of a transfected CHO cells is shown in (C) for reference, but only 10–12 transfected cells were found in an entire 1.5 cm dish per experiment. Incubation overnight with the cholesterol inhibitor M*β* CD (3 mmol/L) facilitates insertion of SGLT1 in the membrane in HEK cells (D) and COS cells (E). In CHO cells, incubation with M*β* CD had no effect (F) and the number of transfected cells remained very low. In (G), the left panels show fluorescence intensity profiles for each condition. The graph in the right panel shows a quantification of SGLT1‐YFP insertion in the plasma membrane, using five different intensity profiles for each condition (see Experimental Procedures for details).

Under normal culture conditions (DMEM), both the YFP and CFP constructs exhibited similar cellular distributions. In HEK cells, a large fraction of SGLT1‐YFP/CFP was in the cytoplasm – likely associated with endosomes – while a small fraction was inserted into the plasma membrane (Fig. 3A). In COS cells, a larger fraction of the SGLT1‐YFP/CFP was located in or at the plasma membrane (Fig. 3B). In CHO cells, there was little or no expression of the constructs (Fig. 3C). We hypothesized in this case that SGLT1‐YFP/CFP's degradation was more rapid than its synthesis. Data are presented later that support this hypothesis.

We next tested the effects of the cholesterol inhibitor M*β*CD on SGLT1‐YFP/CFP internalization in HEK and COS cells. Incubation of HEK and COS cells expressing SGLT1 with 3 mmol/L M*β*CD for 6–12 h caused a significant increase of SGLT1 insertion into the plasma membrane (Fig. 3D and E) (See confocal images in Fig. S2 showing co‐localization of SGLT1 with the plasma membrane marker PMmCherry), suggesting that SGLT1 internalization is lipid raft‐mediated. On the other hand, M*β*CD had no effect on GLUT4‐YFP distribution in either cell type. The transporter remained associated with intracellular compartments, even after a 12‐h incubation in the presence of the inhibitor (result not shown), suggesting that GLUT4 internalization is not lipid raft‐mediated.

**Figure 4:**
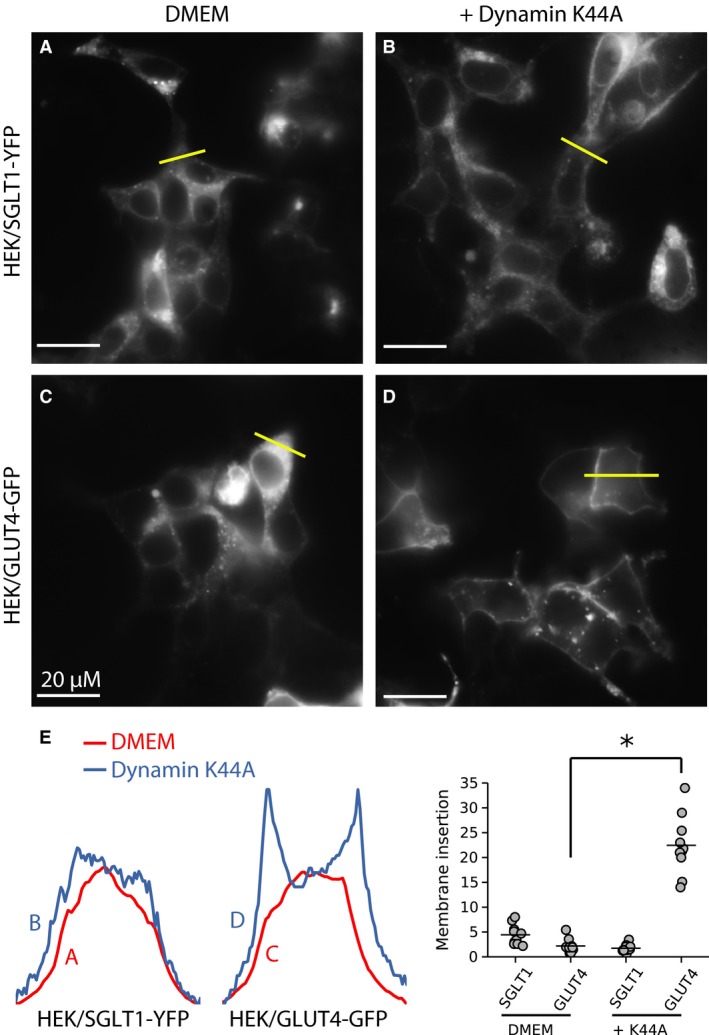
In HEK cells, the dynamin dominant negative K44A facilitates GLUT4 insertion in the plasma membrane, but has no effect on SGLT1 trafficking. Panels A (SGLT1‐YFP expressed in the absence of K44A) and B (co‐expression of SGLT1 with K44A) shows that SGLT1 remains associated with intracellular compartments when co‐expressed with K44A (B). In contrast, GLUT4‐YFP, which is also mostly localized to intracellular compartments under normal conditions (C) is directed to the plasma membrane when co‐expressed with K44A (D). Panel E shows the fluorescence intensity profiles for each condition. The graph in the right panel shows quantifications of SGLT1‐YFP and GLUT4‐GFP insertion in the plasma membrane.

When clathrin‐ or caveolin‐mediated endocytosis was inhibited with overexpression of the dominant negative dynamin K44A, SGLT1‐YFP/CFP insertion in the plasma membrane did not increase (Fig. 4A, B and E), indicating that SGLT1 internalization is not clathrin‐ or caveolin‐dependent. In contrast, under the same conditions, insertion of GLUT4‐YFP into the plasma membrane increased dramatically (Fig. 4C, D and E). This result is consistent with GLUT4 internalization being primarily clathrin‐dependent (Al‐Hasani et al. 1998; Kao et al. 1998; Hou and Pessin 2007; Hou et al. 2009). On this basis, we conclude that internalization of GLUT4 and SGLT1 follow different pathways.

**Figure 5:**
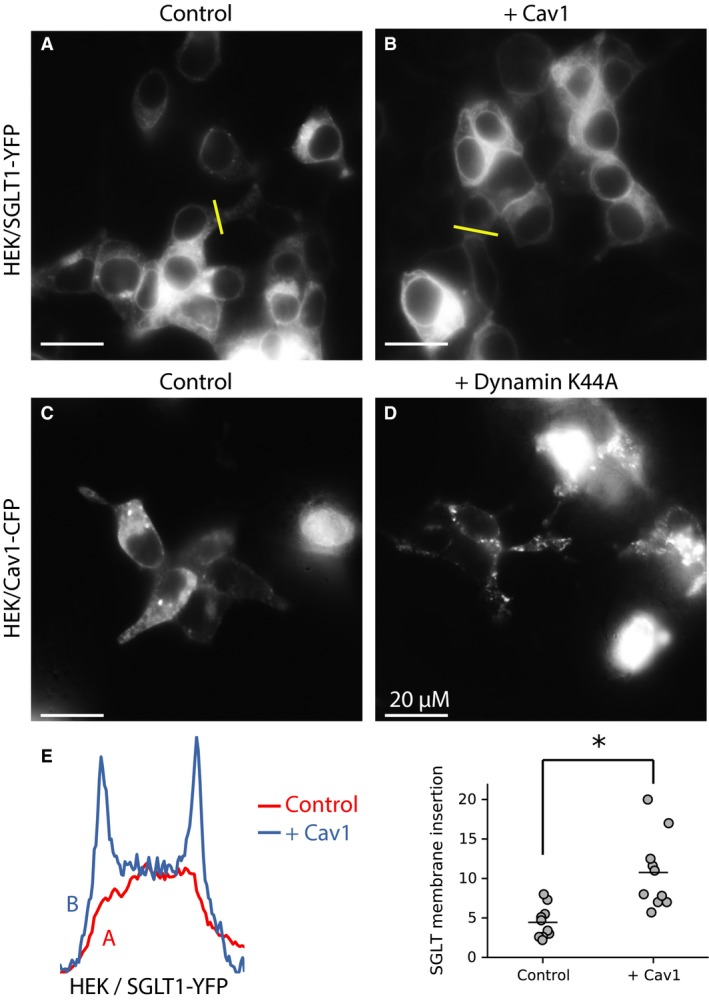
Caveolin 1 (Cav1) does not regulate SGLT1 trafficking in HEK and COS cells. Lipid raft‐dependent endocytosis may be caveolin‐dependent or independent. Overexpression of Cav1 with SGLT1‐YFP in HEK cells causes accumulation of SGLT1 in the plasma membrane (B and E). However, as shown in (F), there is no co‐localization between SGLT1‐YFP and Cav1‐CFP. When expressed alone, Cav1‐CFP is targeted to “hot spots” in the plasma membrane, but is also localized to intracellular compartments (C). When co‐expressed with the dynamin‐dominant negative K44A, there is a strong accumulation of Cav1‐CFP in the plasma membrane (D). These data contrast with that in Panels A and B of Fig 4 where K44A had no effect on SGLT1 trafficking. Panel E shows the fluorescence intensity profiles for (A) and (B). The graph in the right panel shows quantifications of SGLT1‐YFP insertion in the plasma membrane for (A) and (B).

Our dynamin and M*β*CD data indicate that internalization of SGLT1 is lipid raft‐mediated, but not caveolin‐dependent. However, there are reports that overexpression of caveolin 1 (Cav1) increases the transporter's activity by stabilizing SGLT in the plasma membrane (Runembert et al. 2002; Lee et al. 2012; Elvira et al. 2013). To test whether Cav1 had a similar stabilizing effect in our cell systems, we carried out a number of experiments to assess whether (i) the level of Cav1 in the membrane correlates with that of SGLT1‐YFP and Cav1‐mCherry and (Imamura et al. 2009) SGLT1‐YFP co‐localize in the membrane. First, in agreement with others, we found that overexpression of Cav1 increased SGLT1‐YFP insertion into the plasma membrane by 2.5 fold (Fig. 5A, B, and E). However, the increased SGLT1's insertion in the plasma membrane was not associated with accumulation of Cav1 in the plasma membrane. Indeed, the dominant negative dynamin K44A, which prevents Cav1 internalization and increase Cav1 level in the plasma membrane (Achiriloaie et al. 1999; Yao et al. 2005; El‐Sayed and Harashima 2013), had no effect on SGLT1‐YFP insertion in the plasma membrane (Fig. 4B). As a control to test whether dynamin regulates Cav1 internalization in our cell systems, we co‐expressed the dynamin K44A mutant with Cav1‐CFP. Data in Figure 5C and D show that dynamin K44A causes aggregation of Cav1‐CFP in membrane “hot spots”. In addition, incubation of HEK cells with the proteasome inhibitor, MG‐262, which facilitates insertion of SGLT1 in the plasma membrane, had no effect on Cav1‐CFP accumulation in the membrane. Finally, co‐expression of Cav1‐mCherry together with SGLT1‐YFP failed to demonstrate any significant co‐localization of the two proteins (See confocal images in Fig. S3 showing the lack of co‐localization between SGLT1‐YFP and Cav1‐mCherry). These results strongly suggest that there is no correlation between the levels of Cav1 and SGLT1 in the plasma membrane. Thus, SGLT1 stabilization by Cav1 may be indirect and involve intermediary regulatory proteins, such as PI3K or PKC as reported elsewhere (Oka et al. 1997; Zundel et al. 2000).

### Regulation of SGLT1 translocation by metabolic inhibitors

**Figure 6:**
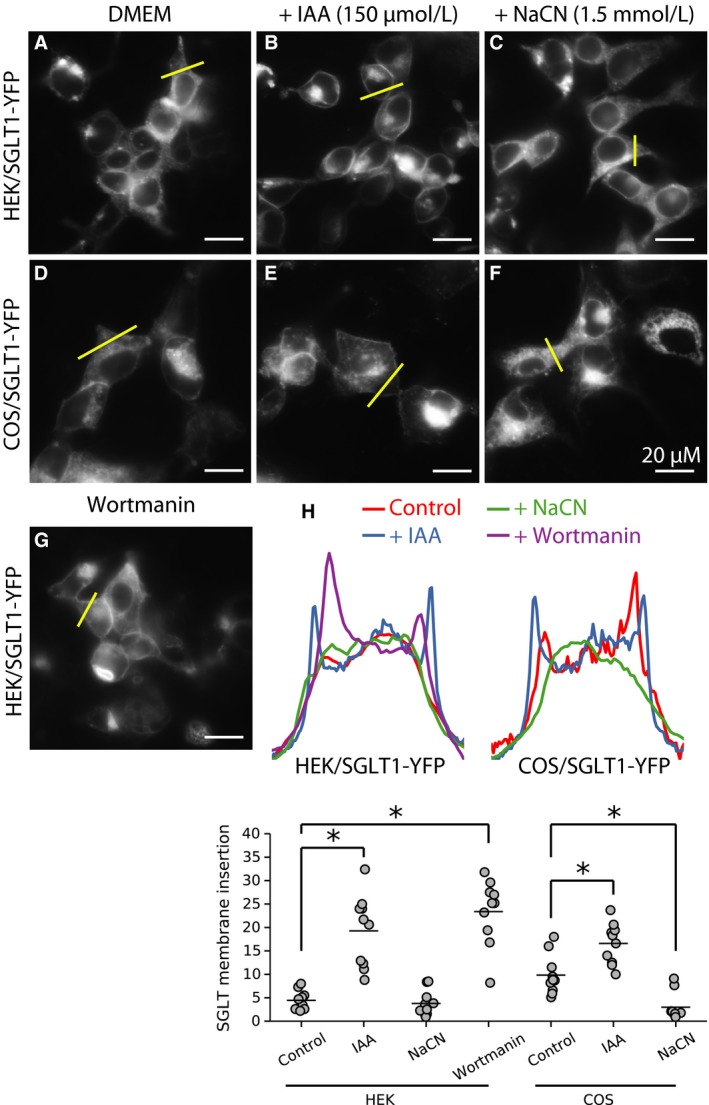
Effects of metabolic inhibitors on SGLT1 trafficking. Vesicle exocytosis and endocytosis are processes that require energy. We tested the effects of inhibitors of ATP production by glycolysis (IAA) and TCA cycle (NaCN) on SGLT1 trafficking. Incubation with 150 *μ*mol/L IAA caused SGLT1 insertion in the plasma membrane within 1–2 h in HEK cells (B) and COS cells (E). In contrast, incubation with NaCN (1.5 mmol/L) had no effect on SGLT1 trafficking in either HEK (C) and COS cells (F). The inhibitor of PI3 kinase, wortmannin causes accumulation of SGLT1 in the plasma membrane (G), suggesting that the effect of ATP is due in part to activation of PI3 kinase. Panel H shows the fluorescence intensity profiles for each of the condition illustrated in Panels (A) to (G). The lower graph shows quantifications of SGLT1‐YFP insertion in the plasma membrane for each condition from (A) to (G).

Membrane endocytosis is regulated by ATP and phosphatidylinositol‐bis 4,5‐phosphate (PIP_2_) synthesis. This ATP/PIP_2_‐dependent process is clathrin‐, dynamin‐, and calcineurin‐independent, but is blocked by M*β*CD (Lariccia et al. 2011). Accordingly, in polarized epithelial cells, the phosphoinositide 3‐kinase (PI3K) inhibitor, wortmannin, increases protein levels in the plasma membrane by preventing their degradation (Hansen et al. 1995; Kjeken et al. 2001). To test whether retrieval of SGLT1 from the plasma membrane is ATP‐dependent, we lowered intracellular ATP with 100 *μ*mol/L iodoacetate (IAA), a glycolytic inhibitor, or 1.5 mmol/L NaCN, a blocker of oxidative phosphorylation. Data in Figure 6 show that IAA promoted accumulation of SGLT1 in the plasma membrane within 6 h (B), but NaCN had no effect even after 12 h (C). Similarly, FCCP, which uncouples mitochondria, had no effect either on SGLT1 trafficking (data not shown). This differential effect of IAA and NaCN or FCCP on SGLT1 translocation probably reflects the greater dependence of these cells on glycolytic rather than oxidative ATP production. To investigate whether this effect of ATP was mediated via PIP_2_ synthesis, we incubated HEK with 1 *μ*mol/L wortmannin. After 6–9 h, there was a significant increase in SGLT1YFP/CFP insertion in the plasma membrane (Fig. 6G), supporting the hypothesis that endocytosis and recycling of SGLT1 is both ATP‐ and PIP_2_‐dependent.

### Degradation of SGLT1 by proteasome and lysosome

**Figure 7:**
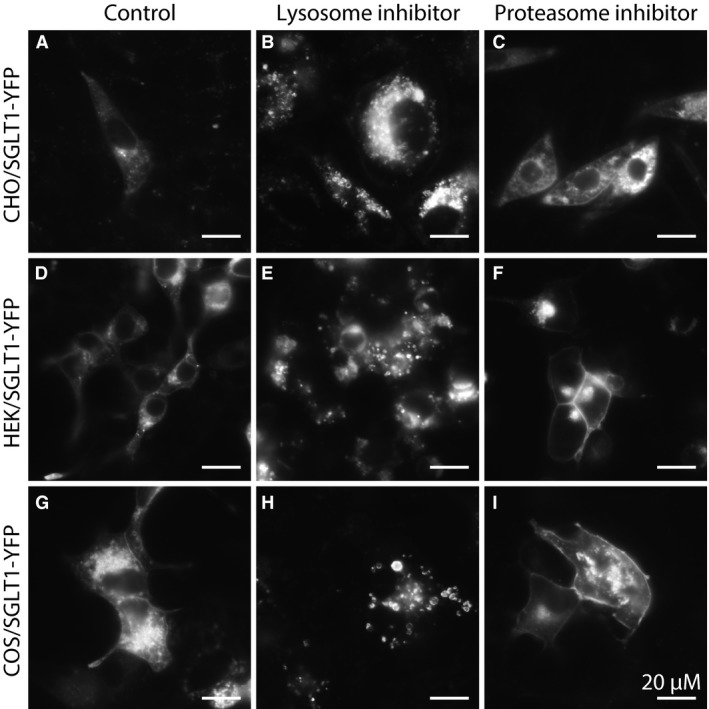
Proteosomal‐ and lysosomal‐dependent degradation of SGLT‐YFP in CHO, HEK, and COS cells. For the series of experiments depicted in this figure, we have used the lysosome inhibitors chloroquine (150 *μ*mol/L) and NH4Cl (10 mmol/L) and the proteasome inhibitors MG262 (1 *μ*mol/L) and lactacystine (5 *μ*mol/L). Panel A illustrates, as previously shown in Fig 3C, that there is almost no expression of SGLT1 in CHO cells under normal culture conditions (A). Addition of the lysosomal inhibitor chloroquine for 4–8 h causes SGLT1‐YFP accumulation in what appears to be the membrane of large vesicles identified as lysosomes (B). Incubation with NH4Cl for the same period of time had a similar, but perhaps lesser effect (not shown). Incubation with the proteasome inhibitor MG262 also causes expression of SGLT1‐YFP in CHO cells, but in this case, a large fraction of the protein was targeted to the plasma membrane (C). Bar graph data in panel (J) indicate that SGLT1 increased insertion of SGLT1 in the plasma membrane in response to MG262 was not associated with increased glucose uptake. The same experiments carried out in HEK and COS cells showed similar results. Incubation with the lysosome inhibitor, chloroquine, directs SGLT1‐YFP to large lysosomal vesicles within 4 h in HEK cells (E) and COS cells (H). Incubation with the proteasome inhibitor MG262 enhanced SGLT1‐YFP insertion in the plasma membrane after 4–6 h in HEK cells (F) and COS cells (I). However, in the cases of HEK and COS cells, most of SGLT1‐YFP was targeted to the plasma membrane (and the Golgi) and there was little fluorescence associated with intracellular compartments. Incubation with the proteasome inhibitor lactacystin had the same effects.

Although CHO cells are routinely used as an expression system for many proteins, including GLUTs, ion channels, hexokinases, and FRET‐based sensors (John et al. 2011), the level of expression of our SGLT1 constructs in these cells was very low. Consistent with this low level of expression, we found that the activity of SGLT1 wt in CHO cells was low compared to that with HEK cells. Low expression of SGLT1 may reflect a lack of protein synthesis or a high rate of protein degradation. Protein degradation may follow one of two pathways: the lysosomal pathway used preferentially by membrane‐bound proteins, or the proteasomal pathway used by cytosolic proteins. To investigate SGLT1 degradation, we first incubated CHO cells expressing SGLT1‐YFP/CFP with the lysosomal pathway inhibitors chloroquine (150 *μ*mol/L) and NH4Cl (10 mmol/L). Within 2 to 4 h following addition of the inhibitors, SGLT1‐YFP/CFP begun to accumulate in lysosomes (Fig. 7B) (See confocal images in Fig. S4 showing SGLT1‐YFP/CFP co‐localization with Lamp1‐mCherry), suggesting that SGLT1‐YFP/CFP is rapidly degraded via the lysosomal pathway. We then incubated the cells with the proteasome inhibitors MG‐262 (1 *μ*mol/L) and lactacystin (5 *μ*mol/L). Again, within 2 to 4 h, SGLT1YFP/CFP expression increased. In this case, however, the fluorescence was associated with the plasma membrane and an intracellular compartment different from lysosomes (Fig. 7C). Even though SGLT1‐YFP/CFP expression could be observed after 2 to 4 h with both either types of inhibitor, the expression level did not reach maximum until 6–9 h. Furthermore, the inhibitors did not have to be present during that time for the expression to reach maximum, in fact a short initial 2‐h incubation was sufficient. Based on these results, we conclude that synthesis and degradation of SGLT1 in CHO cells are fast processes, taking place within hours. Both the lysosomal and proteosomal pathways prevent SGLT1 degradation, even though their effects are dramatically different. To confirm the differential effects of the lysosome and proteasome inhibitors, the same experiments were carried out in HEK and COS cells (Fig. 7D–I). Similar qualitative results were obtained with these cells. In both cases, the lysosome inhibitors chloroquine (150 *μ*mol/L) and NH4Cl (10 mmol/L) induced SGLT1 accumulation in lysosomes after several hours, with the density of the lysosomes increasing during the following 4–6 h. Moreover, as with CHO cells, there was no accumulation of SGLT1‐YFP in the membrane after treatment with lysosome inhibitors (7E and H), whereas accumulation occurred following incubation with proteasome inhibitors. It should be noted that the proteasome inhibitors had slightly different effect in CHO cells and HEK cells. In the latter, the inhibitors did not cause accumulation of SGLT1‐YFP in intracellular bodies, and most of SGLT1‐YFP/CFP was inserted into the plasma membrane (Fig. 7F).

### The respective role of the lysosomal and proteosomal pathways in SGLT1 degradation

**Figure 8:**
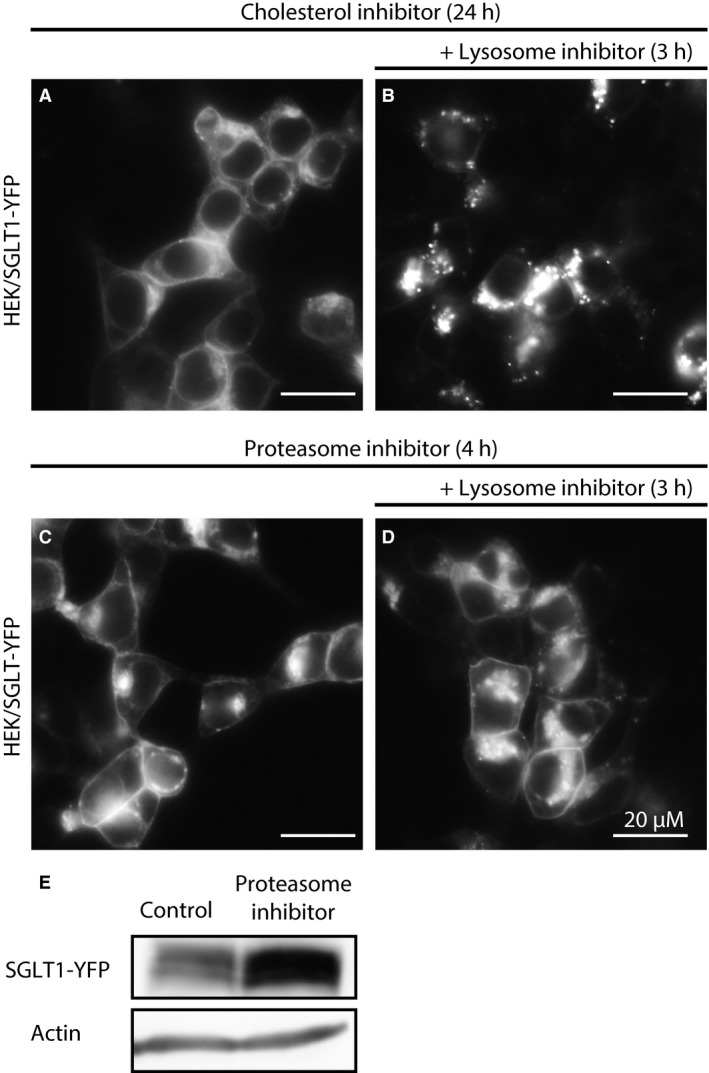
Does SGLT1 traffic via the plasma membrane prior to degradation by lysosomes? To test whether SGLT1 traffics through the plasma membrane prior to be degraded by lysosomes, we first incubated overnight HEK cells expressing SGLT1‐YFP with the cholesterol inhibitor (M*β* CD) (A) and added after that the lysosome inhibitor chloroquine to the incubation medium (B). After incubation with M*β* CD, a large fraction of SGLT1‐YFP is targeted to the plasma membrane (A), but after addition of the lysosome inhibitor, the newly synthesized SGLT1 is directed to lysosome, with no additional insertion in the plasma membrane (B). These data suggest that a large fraction of the newly synthesized SGLT1 is targeted to lysosomes. We did the same experiments using the proteasome inhibitor. In this case, HEK cells expressing SGLT1 were first incubated with MG262 for 4–6 h to target the transporter to the plasma membrane (C), then the lysosome inhibitor choloroquine was added (D). Data in (D) show that, following addition of the inhibitor, SGLT1‐YFP was not targeted to lysosomes, but instead SGLT1‐YFP insertion in the plasma membrane was sustained, while labeling of the Golgi increased. Western blot in (E) shows an increase of 34.5% (*n* = 2) of SGLT1‐YFP protein level after 8 h incubation in the presence of the proteasome inhibitor MG262 (1 *μ*mol/L). Actin (lower band) was used to normalize the protein levels obtained with and without incubation with MG262.

The lysosomal inhibitor data show that SGLT1‐YFP/CFP is mainly targeted to lysosomes for degradation, whereas SGLT1‐YFP/CFP is targeted to the plasma membrane in the presence of proteasome inhibitors. These results raise two questions: (i) Is newly synthesized SGLT1 directly targeted to lysosome for degradation, or is SGLT1 first targeted to the plasma membrane before being degraded by lysosomes; and (ii) is SGLT1 directly degraded by the proteasome, or is the effect of proteasome inhibitors indirect and mediated via inhibition of the lysosomal degradation pathway? To answer the first question, we incubated HEK cells expressing SGLT1‐YFP/CFP with the cholesterol inhibitor, M*β*CD, for 12–24 h to target SGLT1 to the plasma membrane (Fig. 8A), and then added the lysosome inhibitor chloroquine for an additional 3–4 h (Fig. 8B). Few hours after adding the lysosomal inhibitor in the presence of M*β*CD, there was strong labeling of the lysosomal compartment by SGLT1‐YFP/CFP, but no additional insertion into the membrane (Fig. 8B), suggesting that a large fraction of the newly synthesized SGLT1‐YFP is directly targeted to lysosomes for degradation. This is consistent with data obtained with other membrane‐bound proteins where a large fraction of newly formed proteins is directly targeted to lysosomes for degradation, and only a small fraction is targeted to the plasma membrane where it is stabilized and active. The latter fraction is then subsequently recycled to the plasma membrane or to lysosomes for degradation following endocytosis.

We next did the same experiment using a proteasome inhibitor instead of a cholesterol inhibitor. In this case, HEK cells expressing SGLT1‐YFP/CFP were first incubated with the proteasome inhibitor MG262 for 4–6 h (Fig. 8C), and then exposed to the lysosome inhibitor for another 3–4 h (Fig. 8D). In this case, there was no significant targeting of SGLT1‐YFP/CFP to the lysosomal compartment after addition of lysosome inhibitors. In fact, the level of transporter kept increasing in the plasma membrane for the next 6–8 h (Fig. 8D). Consistent with previous reports (van Kerkhof et al. 2001), these results suggest that the proteasome inhibitors block the lysosomal pathway at a late stage. To further test the hypothesis that increased membrane insertion results from blocking of the degradation pathway rather than simple recycling of cytosolic proteins, we carried out western blot analysis to estimate the level of SGLT1‐YFP, before and after a 8 h incubation, in the presence of 1 *μ*mol/L MG262. As shown in Figure 8E, the SGLT1‐YFP level increased by 34.5% (*n* = 2) in the presence of the inhibitor, supporting the hypothesis that this effect is indeed mediated by blocking of the degradation pathway.

## Discussion

We generated SGLT1 fluorescent constructs to study the trafficking of SGLT1‐YFP/CFP in real time in three different cell types. Activity of SGLT1‐YFP/CFP and SGLT1 WT was assessed in parallel experiments, with a FRET‐based glucose sensor. Using live imaging techniques, we observed that translocation of SGLT1‐YFP/CFP is highly regulated and variable depending on the cell type. In HEK cells and COS cells, SGLT1‐YFP/CFP was distributed between intracellular compartments and the plasma membrane, and activity of the transporter depended on the experimental conditions, with SGLT1 activity and insertion in the plasma membrane being higher in COS cells. In contrast, SGLT1YFP/CFP's expression in CHO cells was very low, due to a higher rate of degradation compared to the rate of synthesis. In these cells, inhibition of protein degradation by proteasome or lysosome inhibitors led to SGLT1‐YFP/CFP detection by imaging, but the effects of the two types of inhibitors were dramatically different.

### SGLT1 degradation in CHO and HEK cells

Our data obtained with lysosome and proteasome inhibitors show that both regulate SGLT1‐YFP/CFP expression and trafficking, but in very different ways. Lysosome inhibitors cause accumulation of SGLT1‐YFP/CFP in intracellular bodies, demonstrating SGLT1‐YFP/CFP degradation by lysosomes. In contrast, proteasome inhibitors cause accumulation of SGLT1‐YFP/CFP into the plasma membrane, very likely as a result of lysosomal pathway's inhibition. Furthermore, data obtained after stabilizing SGLT1‐YFP/CFP into the plasma membrane with the cholesterol inhibitor show that addition of lysosome inhibitors still causes rapid accumulation of SGLT1‐YFP/CFP in lysosome, without increasing the level of SGLT1‐YFP/CFP in the plasma membrane. On this basis, we conclude that most of the newly synthesized SGLT1 is targeted to lysosomes for degradation, while a smaller and slower trafficking fraction goes to the plasma membrane. These results are consistent with data obtained with other rapidly cycling membrane‐bound proteins such as connexin43 (Cx43) (Laing et al. 1997; Qin et al. 2003). In comparison, data obtained with proteasome inhibitors were somewhat less expected. Indeed, in all three cell lines, the inhibitors caused accumulation of SGLT1‐YFP/CFP into the plasma membrane. In CHO cells, this effect was accompanied by accumulation of SGLT1‐YFP/CFP in intracellular bodies, but in HEK cells, SGLT1‐YFP/CFP was found in the plasma membrane and in the Golgi, but not in intracellular bodies. These effects of the lysosome and proteasome inhibitors on SGLT1‐YFP trafficking are very reminiscent of the effects on Cx43, wherein lysosome inhibitors caused accumulation of the protein in intracellular bodies, while proteasome inhibitors, induce accumulation in the membrane. How proteasome inhibitors cause Cx43 insertion into the membrane still remains largely unknown.

Studies of inhibition of EGFR and GHR degradation by proteasome inhibitors may be a key to understanding our SGLT1 data. It has been shown that inhibition by proteasome inhibitors of a late step in the lysosomal pathway – between the late endosome and lysosomes – increases recycling of EGFR and GHR to the plasma membrane (van Kerkhof et al. 2001; Longva et al. 2002; Alwan et al. 2003; Lipkowitz 2003). Similar results obtained with SGLT1‐YFP/CFP suggest that the increased membrane insertion of SGLT1 induced by proteasome inhibitors may result from inhibition of the lysosomal degradation pathway at a late stage, which in turn favors recycling of SGLT1 from the early endosome to the plasma membrane. Such a mechanism would explain our data obtained with HEK cells where proteasome inhibitors cause accumulation of SGLT1 in the plasma membrane, while preventing at the same time its accumulation in intracellular bodies. Indeed, it is logical to assume that the inhibitors block a step – that is, the late endosome to lysosome step – which is common to both the TGN to the lysosomes pathway, denoted A in Figure 2, and the plasma membrane to the lysosome pathway denoted B in Figure 2. Furthermore, our observation that SGLT1 protein levels increase by 34.5% after incubation with the proteasome inhibitor, is also consistent with inhibition of the lysosomal degradation pathway. Using an HA‐tagged ubiquitin, we were not able to show that SGLT1 itself is ubiquitinated (pers. obser.), suggesting that the proteasome inhibitor does not have a direct effect on the transporter endocytosis and degradation. However, this does not invalidate our hypothesis, since the proteasome may target the transfer process of the cargo proteins rather than the proteins themselves (van Kerkhof et al. 2001; Longva et al. 2002).

### SGLT1 internalization in HEK and COS cells

It has been suggested, based on the observation that SGLT1 activity is upregulated by caveolin, that caveolin increases insertion of the transporter in the plasma membrane (Runembert et al. 2002; Lee et al. 2012; Elvira et al. 2013). Our data support this hypothesis and show that co‐expression of SGLT1YFP/CFP with caveolin 1 (Cav1) causes a significant increase in the insertion of SGLT1‐YFP/CFP in the plasma membrane. The best known role of caveolin is that of a scaffolding protein, which organizes and inactivates signaling molecules that are concentrated on the cytoplasmic surface of caveolar membranes. Such inactivation of membrane‐bound proteins cannot account for the stimulatory effect of caveolin on SGLT1. Then, the role of caveolin is not restricted to that of a scaffolding protein as it may also interact and regulate the activity of signaling molecules, including G proteins, adenylate cyclase, PKC, or the insulin receptor (Smart et al. 1999; Ishikawa et al. 2005). We carried out experiments to further investigate the regulation of SGLT1 activity by caveolin. As already stated, co‐expression of SGLT1‐YFP/CFP with wild‐type Cav1 increases the insertion of SGLT1 in the plasma membrane. However, when SGLT1‐YFP was co‐expressed with Cav1‐mCherry, we could not find any overlap in the plasma membrane (Fig. S3). These data indicate that the effect of Cav1 on SGLT1 insertion in the plasma membrane is indirect and may involve PKA, which is regulated by cytoplasmic caveolin (Lee et al. 2012), PI3K or PKC as reported elsewhere (Oka et al. 1997; Zundel et al. 2000). To further rule out a direct effect of caveolin on SGLT1 insertion in the plasma membrane, we tested the effect of the dominant negative dynamin K44A, which stabilizes caveolin in the plasma membrane (Henley et al. 1998; Oh et al. 1998; Smart et al. 1999; Parton and del Pozo 2013). Accordingly, our data obtained with HEK and COS cells show that expression of dynamin K44A causes caveolin accumulation in membrane domains. However, this effect of dynamin K44A on caveolin occurs without a parallel increase in membrane‐bound SGLT1‐YFP, further supporting the hypothesis that regulation of SGLT1 trafficking by caveolin is indirect and does not require caveolin's stabilization in the membrane.

Thus, SGLT1 internalization may be lipid raft‐dependent, but caveolin‐independent. A number of pathways have been identified that are cholesterol‐mediated, but caveolin‐independent. For instance, macropinocytosis is clathrin and dynamin‐independent, but is regulated by M*β*CD. In addition to be regulated by M*β*CD, this pathway is controlled by PIP2 and the GTPase Cdc42, which controls actin polymerization (Lariccia et al. 2011). We found that SGLT1 internalization is modulated by the inhibitor of PI3K wortmannin, but is insensitive to overexpression of the dominant negative Cdc42N17 (data not shown). These results indicate that SGLT1 endocytosis is not mediated via macropinocytosis. There are other pathways that are lipid raft‐dependent and caveolin‐independent, such as the flotillin‐dependent pathway, or the Arf6‐dependent pathway (El‐Sayed and Harashima 2013), which may regulate SGLT1 internalization. Future experiments will have to be carried out with RNAi against flotillin and Arf6 to test for the possible role of these other cholesterol‐dependent pathways in SGLT1 translocation.

### Physiological relevance: A model for polarized epithelial cells

**Figure 9:**
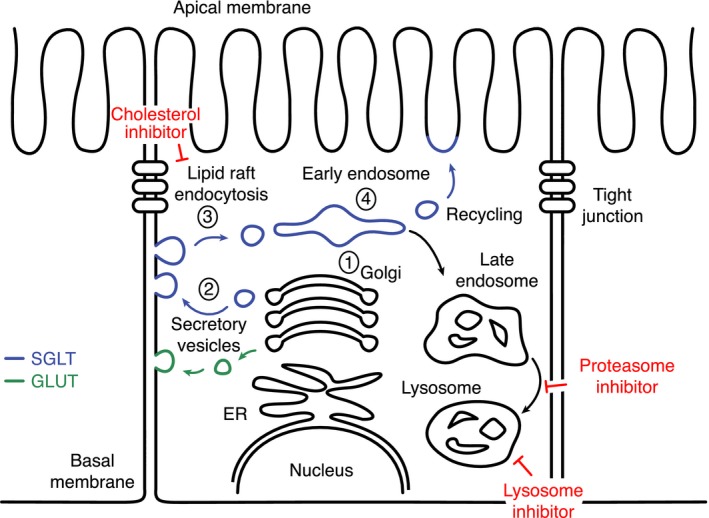
Model for SGLT1 and GLUT4 trafficking in polarized epithelial cells. Based on our data as well as those of others and the model of Rodriguez‐Boulan (Rodriguez‐Boulan et al. 2005; Lakkaraju and Rodriguez‐Boulan 2007), we propose that SGLT1 traffics from the Golgi (1) to the basolateral membrane (3) before being internalized to endosome (4) and finally inserted into the apical membrane. This trafficking pathway has been denoted the transcytotic pathway, in opposition to the direct pathway that takes the proteins from the Golgi to the apical membrane. In this model, we assume that the basolateral to apical membrane trafficking is lipid‐raft mediated. It follows based on this simple assumption that SGLT1, which internalization is cholesterol‐dependent, would translocate to the apical membrane, while GLUT4, which internalization is not cholesterol‐mediated, would remain in the basolateral membrane.

In MDCK cells, SGLT transits from the whole cell membrane to the apical membrane as tight junctions form and the cells become polarized. Exposure of these polarized cells to the cholesterol inhibitor M*β*CD causes SGLT1 depletion from the apical membrane and accumulation into the rest of the membrane (Suzuki et al. 2006). How cholesterol regulates SGLT1 membrane targeting in these polarized cells remains unclear. Our data obtained with HEK and COS cells exposed to M*β*CD show that cholesterol depletion causes accumulation of SGLT1 in the plasma membrane, suggesting that in the absence of cholesterol, SGLT1 endocytosis is inhibited. This accumulation of SGLT1 in the plasma membrane in response to M*β*CD is consistent with reports obtained with other proteins, including cholera toxin and GLUT transporters (Ivanov 2008). In epithelial cells, protein targeting to the apical membrane may occur via a direct route from the Golgi to the apical membrane, or a transcytotic route with a stopover at the basolateral membrane (Rodriguez‐Boulan et al. 2005; Lakkaraju and Rodriguez‐Boulan 2007). Based on this model, our M*β*CD data are best explained, assuming that in polarized epithelial cells, SGLT1 targeting to the apical membrane proceeds with a stopover at the basolateral membrane where regulation of trafficking by cholesterol synthesis takes place (see Fig. 9). Under normal conditions, SGLT1 would be transiently inserted into the basolateral membrane, on its way to the apical membrane, but following membrane depletion of cholesterol, endocytosis of SGLT1 at the basolateral membrane would be inhibited and SGLT1 would accumulate in the basolateral membrane. As a consequence of inhibition of this forward traffic, there would be depletion of SGLT1 from the apical membrane. When comparing the effects of different modulators of endocytosis and degradation, we found that regulation of SGLT1 and GLUT4 trafficking was different. Indeed, we found, consistent with clathrin‐dependent internalization of GLUT4 (Al‐Hasani et al. 1998; Kao et al. 1998; Hou and Pessin 2007; Hou et al. 2009), that internalization of GLUT4 was M*β*CD‐insensitive and dynamin‐dependent. Based on these observations, we propose the model depicted in Figure 9 where the transcytotic pathway between the basolateral membrane and the apical membrane is lipid raft‐mediated; with this assumption alone we can explain SGLT1 preferential targeting to the apical membrane and GLUTs targeting to the basolateral membrane.

### Stress‐related expression of SGLT1 in muscle

In cardiac muscle, SGLT1 is chronically upregulated during ischemia and in diabetes (Banerjee et al. 2009) and its overexpression causes hypertrophy (Ramratnam et al. 2014). In addition to these chronic effects, increased activity of SGLT1 occurs acutely, within 1 h, in response to leptin and insulin (Banerjee et al. 2009). Similarly, in skeletal muscle, the activity of SGLT1 increases within 1 h in response to exercise (E.M. Wright personal communication). The chronic effects of diabetes and ischemia are best explained by increased gene expression, but there is no clear understanding of the mechanism of acute upregulation of SGLT1. This rapid increase in activity may result from insertion of premade transporter into the plasma membrane, or as supported by our CHO trafficking data, from increased expression of SGLT1 after inhibition of protein degradation due to physiological or pathological stress. This hypothesis, which is consistent with the well‐known upregulation of ubiquitin ligases by ER stress (Shen et al. 2007) is presently being tested in our laboratory, using isolated muscle fibers and the FRET‐based glucose sensor.

In summary, in mammalian cells, newly synthesized SGLT1 may be targeted to lysosomes for degradation, or directed to the plasma membrane for glucose transport. Once in the plasma membrane, SGLT1 can be internalized via a lipid raft‐dependent process to be either recycled to the plasma membrane, or directed to lysosomes for degradation under control of the proteasome. In contrast, GLUT4 internalization is lipid raft‐independent, but degradation involves lysosomes. Having established how SGLT and GLUT are internalized and degraded in nonpolarized cells, it would be relevant to determine how the processes, which we characterized, influence the targeting of these transporters to the apical and basolateral membrane in polarized cells.

## Conflict of Interest

None declared.

## Supporting information




**Fig S1.** SGLT1 and SGLT2‐dependent glucose transport measured in COS cells, expressing the FRET‐based glucose sensor Flip600*μ*M together with either wt SGLT1 or wt SGLT2.
**Fig S2.** Confocal images of the Plasma Membrane marker‐mCherry (PMmCherry) (left panel) and SGLT1‐YFP after incubation with the proteasome inhibitor MG‐262 (1 mM) (middle panel). The right panel is an overlap of images A and B.
**Fig S3.** Confocal images of punctate Cav1‐mCherry at the cell periphery (left panel) and of membrane‐inserted SGLT1‐YFP, after incubation with the proteasome inhibitor MG‐262 (1 mM) (middle panel). The right panel shows no overlap between Cav1‐mCherry and SGLT1‐YFP.
**Fig S4.** Confocal images of Lamp1‐mcherry, labeling a dense pattern of lysosomes throughout the cytoplasm (left panel), and of SGLT1‐YFP, labeling a less dense punctate pattern (middle panel). The overlap of images A and B (right panel) shows that SGLT1 is targeted to some lysosomes (arrows).
