# Supplementary material for: Real‐time imaging of sodium glucose transporter (SGLT1) trafficking and activity in single cells
Source: Physiol Rep. 2017 Feb 13;5(3):e13062. doi: 10.14814/phy2.13062 (PMC5309568; doi:10.14814/phy2.13062)
Supplement: Supplementary file 1 — Fig S1. SGLT1 and SGLT2‐dependent glucose transport measured in COS cells, expressing the FRET‐based glucose sensor Flip600μM together with either wt SGLT1 or wt SGLT2. Fig S2. Confocal images of the Plasma Membrane marker‐mCherry (PMmCherry) (left panel) and SGLT1‐YFP after incubation with the proteasome inhibitor MG‐262 (1 mM) (middle panel). The right panel is an overlap of images A and B. Fig S3. Confocal images of punctate Cav1‐mCherry at the cell periphery (left panel) and of membrane‐inserted SGLT1‐YFP, after incubation with the proteasome inhibitor MG‐262 (1 mM) (middle panel). The right panel shows no overlap between Cav1‐mCherry and SGLT1‐YFP. Fig S4. Confocal images of Lamp1‐mcherry, labeling a dense pattern of lysosomes throughout the cytoplasm (left panel), and of SGLT1‐YFP, labeling a less dense punctate pattern (middle panel). The overlap of images A and B (right panel) shows that SGLT1 is targeted to some lysosomes (arrows). [file PHY2-5-e13062-s001.pdf]

SUPPLEMENTAL FIGURES

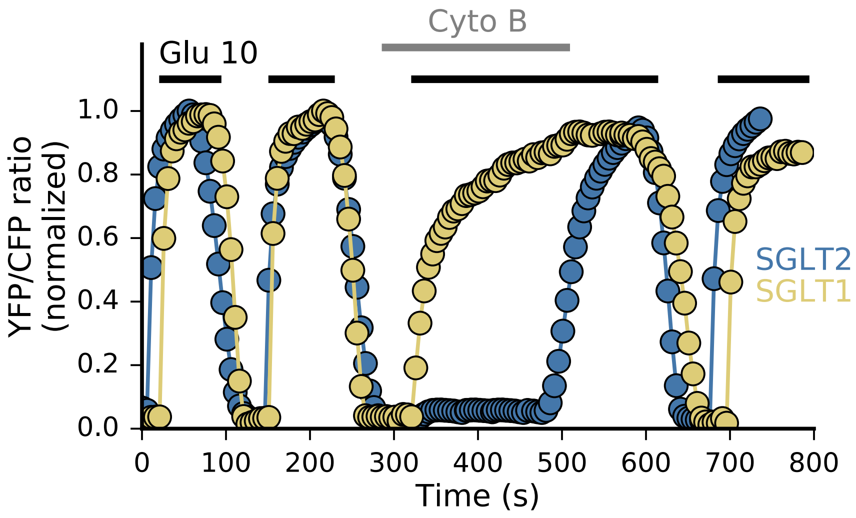

Figure S1. SGLT1 and SGLT2-dependent glucose transport in COS cells. This panel shows a superimposition of two traces obtained in two sets of experiments with COS cells expressing the FRET-based glucose sensor Flip600μM together with either wt SGLT1 or wt SGLT2. These traces demonstrate that the glucose uptake mediated via SGLT2 is only a fraction of that mediated via SGLT1. This data is consistent with previously published results (Hummel, Lu et al. 2011.

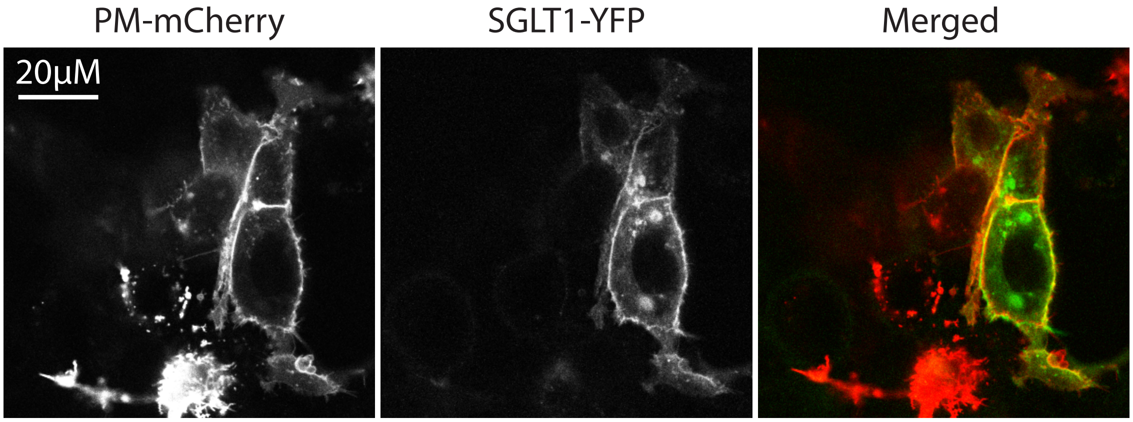

Figure S2. Confocal images showing colocalization of the Plasma Membrane marker-mCherry (PM-mCherry) and SGLT1-YFP in HEK cells. Overexpression of PM-mCherry nicely delineates the plasma membrane (left panel). Similar labeling is observed with SGLT1-YFP (middle panel) after incubation with the proteasome inhibitor MG-262 (1 μM). Overlap of images A and B (right panel) shows that SGLT1-YFP is indeed inserted in the membrane. Similar overlap was observed following HEK cells incubation with the cholesterol inhibitor MβCD (3 mM).

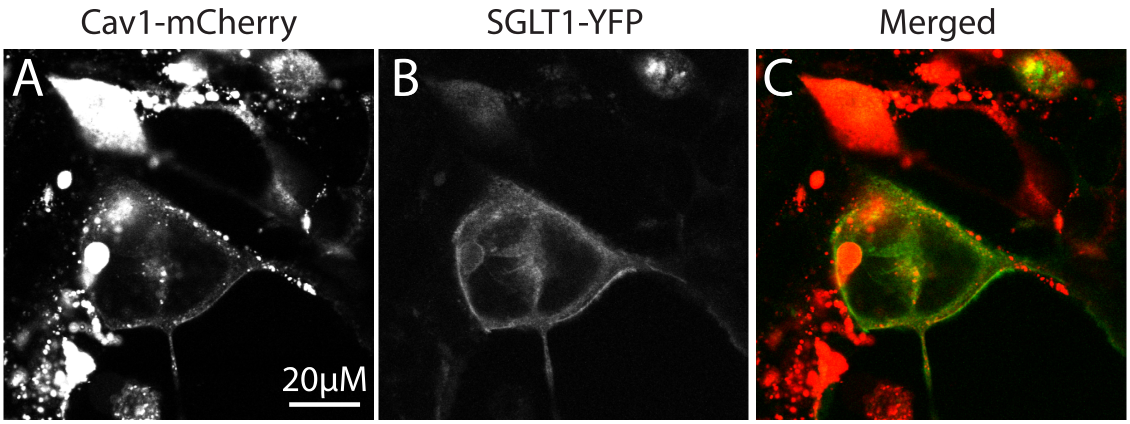

Figure S3. Confocal images showing that overexpression of Cav1-mCherry yields punctuated fluorescence at the cell periphery, whereas overexpressed SGLT1-YFP is targeted to the plasma membrane (middle panel) after incubation with the proteasome inhibitor MG-262 (1 μM). The right panel shows the lack of overlap between Cav1-mCherry and SGLT1-YFP. These data indicate that increased insertion of SGLT1in the plasma membrane occurs without direct interaction with Cav1.

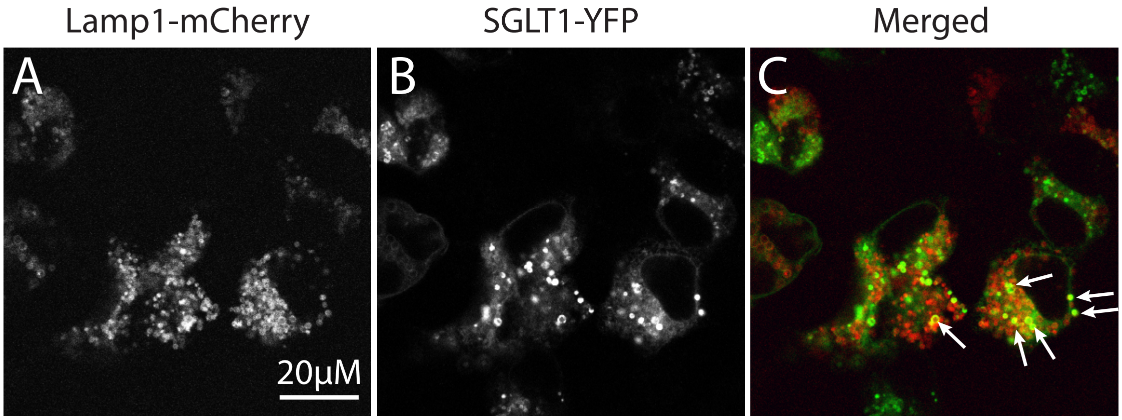

Figure S4. Confocal images showing the colocalization of Lamp1-mCherry and SGLT1-YFP in HEK cells. Overexpression of Lamp1-mcherry labels a dense pattern of lysosomes throughout the cytoplasm (left panel). Similar labeling, although of lesser density, is observed with SGLT1-YFP (middle panel). Overlap of images A and B (right panel) shows that SGLT1 is targeted to some, but not all, lysosomes (arrows).
